# Resonance in recovery: group singing as a mechanism for collective narrative repair, emotional integration, and post-traumatic growth

**DOI:** 10.3389/fpsyg.2025.1763648

**Published:** 2026-01-12

**Authors:** Yichang Liu

**Affiliations:** Luxun Arts School, Yan'an University, Yan'an, China

**Keywords:** arts-based intervention, choir, group singing, narrative repair, polyvagal theory, posttraumatic growth, trauma recovery

## Abstract

Psychological trauma often disrupts both bodily regulation and personal narrative, making it hard for survivors to put their experience into words and to stay connected with others. Traditional talk therapies rely on verbal storytelling, but early or pre-verbal trauma, fragmented memories, and strong shame can limit what language alone can reach. This paper proposes choir-based group singing as a form of collective narrative repair. Drawing on polyvagal theory, we describe how stable breathing and gentle vocalization in choral singing can signal safety to the nervous system, lower arousal, and reshape stress-related physiological responses. We then examine how nonverbal vocal expression, shared lyrics, and aesthetic distance allow trauma-related emotions to be expressed and contained without direct disclosure. Harmonic structure, voice blending, and bodily synchrony provide an external order for fragmented experience and support a shift from a victim-centered identity toward roles such as singer and collaborator. The paper further links joint intentionality in rehearsal and performance to reconstruction of meaning and posttraumatic growth, as survivors act with others toward shared musical goals and regain a sense of belonging. Finally, we highlight methodological limits in current empirical work and outline directions for interdisciplinary research that bridge psychotherapy, music education, and social science. Taken together, the analysis suggests that choir is not only an emotional outlet but a structured, relational practice that can help survivors rebuild safety, story, and connection.

## 1 Introduction

Psychological trauma changes how the mind and body work. When people recall traumatic events, activity in brain regions for language often goes down, while activity in regions linked to emotion and arousal becomes stronger. This pattern has appeared in several neuroimaging studies. For example, functional MRI studies have shown that language-processing areas are suppressed during traumatic recall (Rauch et al., 1996). Bessel van der Kolk also argues that people often cannot put trauma into words because the body stores the experience in its own way (der Van Kolk, 2014). These findings suggest that many traumatic memories do not exist in a form that can be easily spoken. They appear as bodily sensations, emotional surges, or fragmented images. People find it hard to explain these experiences in everyday language, so they may feel out of control and withdraw from social life.

Traditional talk therapies have clear limits here. They ask people to tell their stories in words. However, many studies suggest that traumatic events that occur very early in life, or before language is fully developed, often lack a verbal structure and are hard to turn into a coherent narrative (Herman, 1992; Ogden et al., 2006). Because of this, talking alone is often not enough for people to work through their deepest experiences. They need ways for the body and emotions to take part in the healing process. Arts-based interventions have drawn growing attention in response to this need.

Choral singing is a typical form of arts-based intervention. It brings people together to use their voices in a shared and safe group setting. They breathe together and move with the same rhythm. A number of studies show that group singing can reduce stress, strengthen social bonds, and increase emotional stability. For example, research has found that choir participation improves mood and social experience (Fancourt and Perkins, 2018). Other work suggests that collective singing can make it easier for people to feel with and for others (Pearce et al., 2015). Recent empirical work has begun to quantify these effects using psychobiological markers. For example, in a controlled comparison of leisure activities among Japanese older adults, choir singing was associated with a decrease in salivary cortisol, whereas a cognitively engaging control activity (go playing) showed an increase, suggesting that singing may down-shift HPA-axis arousal in a socially embedded format (Shoda et al., 2025). Complementary evidence from a 12-week group-singing program also reports pre–post session reductions in salivary cortisol and alpha-amylase, consistent with acute stress-regulatory effects of collective singing (Mallik et al., 2025). These findings indicate that choral singing is not only a musical activity. It is an experience that can help regulate bodily rhythms, emotional states, and social relationships.

In this context, collective narrative repair is the central concept proposed in this paper. This concept focuses on how people can find their own voice again in a group setting. Vocal exchange in a choir does not depend on language. It allows participants to express inner experiences through melody, breath, and bodily feeling. When people who have gone through trauma sing in a choir, they do not need to find the right words first. They can still be heard and supported by others. As the group’s breathing becomes more synchronized, the rhythm steadies, and support continues over time, these singers may slowly link their scattered experiences together. This process may open up new pathways for growth after trauma.

## 2 The resonant body: non-verbal expression and emotional integration

People often feel relaxed, steady, and supported when they sing. Work in neurobiology helps explain this experience. Polyvagal theory proposes that the human autonomic nervous system shifts back and forth between states of safety and defense, and that the ventral vagal pathway is a key route for this regulation. Singing relies on steady breathing and soft vocal production. These actions can engage the ventral vagal system. Researchers argues that a stable breathing rhythm and warm vocal tone send the brain a signal that the situation is safe, which lowers physiological arousal (Stephen and Eichhorn, 2017). For many people who have lived through trauma, the sense of calm that appears during singing is therefore not just a random emotional change. It reflects how the body responds to clear signals of safety. Importantly, emerging clinical-context studies suggest that psychobiological effects may be context-sensitive. In a pilot protocol delivered in child and adolescent psychiatry settings, short-term arts activities including choir singing were feasible and associated with improvements in mood-related outcomes, while biomarker changes were more mixed—highlighting the need to specify dosage, setting, and measurement timing when theorizing stress-regulation mechanisms (Grebosz-Haring and Thun-Hohenstein, 2024).

Singing can also offer a nonverbal way for trauma survivors to express themselves. Many survivors struggle to describe their suffering in words, because traumatic memories often take the form of bodily sensations or mental images rather than a clear story. Singing gives these unspoken emotions a safe channel. When a person holds a note for longer, shapes a melodic phrase, or adds more breath and force at a certain point, their body is expressing feelings that may not yet have words. Herman argues that healing from trauma often requires forms of expression that come before language (Herman, 1992). In a choir, people do not have to explain their trauma in detail. They can let the voice carry their emotions and let the melody serve as a path for those emotions to move. Rather than reconstructing a coherent story at this stage, singing offers a graded way to access, hold, and release affect through breath, phrasing, and vocal intensity.

This kind of expression happens not only at the level of the individual but also at the level of the group. Studies show that when people sing together, their breathing patterns and heart rates tend to become synchronized. Müller and Lindenberger recorded synchronized heart rate variability among choir members, which suggests that their bodies enter a shared rhythm (Müller and Lindenberger, 2011). Breathing shows a similar pattern. When everyone inhales at the same point in the phrase and exhales along the same rhythmic line, the choir develops a physical sense of resonance. This resonance is not just a metaphor. It is a real form of bodily synchrony. It allows participants to feel that they are not isolated individuals but part of a group that breathes and voices together.

This physiological synchrony gives a concrete base for emotional integration. When the bodily rhythm of a trauma survivor can align with the rhythm of others, it can become easier for that person to feel accepted and to trust others. In choral singing, shared sound, shared rhythm, and linked breathing support people in moving out of isolation. They can locate themselves again within a network of safe relationships. In this way, nonverbal expression in singing is not only a release of emotion. It is also a process through which the body can learn a new sense of safety. In this section, the emphasis is on physiological safety and affect regulation—how choir makes trauma-related emotions more tolerable in the body—before explicit meaning-making or life-story integration is possible.

## 3 Re-storying in harmony: choir as collective narrative repair

Building on the regulatory foundation described in section 2, this section shifts from affect to meaning: how musical structure and shared lyrics can scaffold narrative coherence and identity repair. Trauma can create deep breaks in a person’s sense of self and life story. Many survivors find that when they try to describe what happened, their story feels disjointed and their identity seems split into many pieces. Work in psychiatry often refers to this as narrative fragmentation and notes that trauma can weaken the capacity to link past, present, and future into a coherent whole (Herman, 1992). When the story cannot hold together, it becomes hard to find meaning in what has been lived. The musical structure of choral singing offers an external order that can meet this broken state. Multi-part harmony gives the song a layered, stable, and predictable form. As people sing within this structure, they naturally feel that they are stepping into a complete frame. When one voice joins another and finds its place in the harmony, the singer also experiences a sense that they can still be in tune with the world. This experience can lay the groundwork for later repair of personal narrative.

Many choral texts speak of shared human feelings such as sadness, loss, hope, or perseverance. These emotions are not the private history of one individual. They are experiences that the whole group can understand. Here, lyrics and musical form function as a semantic scaffold: they provide a shared sequence and interpretive frame that can later be mapped onto personal experience as narrative coherence returns. Psychologists have described this as a form of alternative narrative, where people find themselves in someone else’s story and use that indirect route to sort through their own inner states (White and Epston, 1990). In this way, choir can support meaning-making without requiring immediate autobiographical completeness.

Choral singing is also a decentered way of expression. In solo singing, every sound comes from a single person, which can feel demanding or overwhelming for someone who has been through trauma. In a choir, by contrast, each individual voice is held inside a larger field of sound. The singer is no longer trapped in the pair of me and my pain, but becomes part of a musical whole. Research suggests that post-traumatic shame often arises from a strong self-focused perspective and that this shame tends to keep people silent (Dorahy et al., 2017). The blending of voices in choir can ease this burden of the self, because the person starts to sense that they are not a solitary victim. They are joining others to express a shared emotional theme. This kind of experience can soften shame and make it easier to move back into social relationships. Consistent with these proposed pathways, recent longitudinal and mixed-methods work in inclusive community-choir contexts reports gains in emotional wellbeing, self-esteem, and belonging, alongside reductions in anxiety- and depression-related symptoms, with participants explicitly describing the choir as a safe space for emotional expression and relational reconnection (Juan-Morera et al., 2024).

Choral work also depends on joint intentionality. Each member of the choir must attend to the point of inhalation, the shape of the phrase, and the stability of the pulse. People work toward shared goals, such as cleaner harmony, steadier tempo, or more expressive phrasing. Psychological research suggests that when people act for a common purpose, they feel that they are taking part in something meaningful (Wolf and Tomasello, 2025). For trauma survivors, this feeling is crucial, because trauma often leaves them with the sense that their ties with others have been broken. When they share attention, goals, and musical outcomes with other singers, they are also rebuilding a felt sense that they are working together to mend something. This kind of experience is itself a narrative act, because it places the person back into a story in which they can be seen, heard, and supported by others.

## 4 From isolation to connection: social support and PTG

Trauma often pulls people away from social life. Many survivors cut back on contact with others because they worry that no one will understand what they have been through, or they fear being hurt again. They may also feel as if they no longer live in the same world as the people around them. This state increases isolation and weakens their ability to recover. A choir offers an easy way back into a social setting. When people come to rehearsal, they do not have to start conversations or show anything about themselves. They can simply follow the sound of the group and sing along. This quiet corner of social life can feel safe for those who tend to withdraw and can slowly help them return to a shared space.

As a person starts to find a place in the choir, their sense of identity can also begin to shift. Many trauma survivors feel strongly defined by the word victim. This label often comes with shame and a sense of helplessness. In a choir, no one is fixed in that role. When someone learns their line, keeps up with the rhythm, or completes a musical phrase, they see that they hold a different role in this setting. They may start to think of themselves as a singer, a choir member, or a person who can work with others. This change in identity can bring new strength and make it easier to build connections.

Moving and sounding in sync helps people feel close more quickly. Researchers have noted that bodily synchrony can increase trust and prosocial behavior. Experiments show that when people sing together or perform the same actions at the same time, they are more willing to cooperate and feel more friendly toward each other (Cross et al., 2019). In choir, synchrony is especially strong, because members must match their breathing, rhythm, and pitch. In this shared timing, people often experience a sense of we, which is a key base for rebuilding relationships after trauma.

Choral singing can also support posttraumatic growth. This process involves several aspects. As people learn vocal skills or master new musical passages, they experience a sense of self-efficacy. Bandura argues that when people feel able to handle a task, their inner sense of power increases (Bandura, 1977). Many trauma survivors doubt their own abilities. Successful experiences in music can break this pattern and offer a different view of what they can do.

Choir work can change how people experience others as well. When someone sings in an ensemble, they need to listen to the other parts. If they sing too loudly, the harmony becomes unbalanced. If they hold back too much, the sound becomes thin. This mutual dependence teaches people how to notice and value others. Studies suggest that artistic collaboration can increase sensitivity to others and appreciation of their contributions (Lauss et al., 2024). In a choir, people can feel the value of others through the way their voices blend.

Music can also bring changes at a spiritual level. Many choral pieces carry feelings of solemnity, sacredness, or transcendence. When people sing this kind of music, they often experience a sense of calm or meaning that goes beyond daily life. In their theory of posttraumatic growth, Tedeschi and Calhoun note that after major trauma some individuals find a new direction in life through spiritual experience (Tedeschi and Calhoun, 2004). The emotional atmosphere of choral singing can hold such experiences and give people a musical way to reconsider their life story.

Taken together, social connection, bodily synchrony, and emotional expression in choir can help survivors move from isolation toward a sense of belonging. They are no longer carrying their pain alone. Through shared sound, they begin to form new identities and new meanings. These shifts create conditions under which posttraumatic growth becomes possible.

## 5 Discussion and future directions

Existing research on choir participation and trauma recovery offers important clues, but it also has clear limits. Many studies include small samples and rely mainly on interviews or observational reports. These methods help us understand how people experience choir, but they make it hard to test causal effects. Future research would benefit from designs that can separate choir-specific mechanisms from non-specific group effects. A pragmatic approach is a longitudinal trial with an active comparator, assessed across multiple timepoints to model change trajectories rather than single pre–post differences. Standardized outcomes could include PTSD symptom severity, social connectedness and belonging, and posttraumatic growth, complemented by mechanism-focused measures such as heart rate/heart rate variability and salivary stress markers. Given evidence that optimal PCL-5 cutoffs may vary by population and purpose, symptom trajectories and continuous outcomes may be more informative than categorical screening alone. Psychophysiological data should be collected and reported with contemporary methodological guidance, explicitly controlling key influences to improve interpretability and reproducibility.

The mechanisms of choir’s effects also need more detailed work. We know that group singing can support emotion regulation and social connection, but we do not yet know what it adds beyond other group activities. For example, team sports and group dance can also produce synchrony and group identity, and group psychotherapy can offer structured meaning-making through language. However, choral singing uniquely combines (a) sustained breath–voice coupling, (b) semantic scaffolding through shared lyrics, and (c) acoustic blending that requires moment-to-moment attunement to others. This combination may make choir especially suited to trauma-informed work where direct disclosure is difficult, yet regulation, expression, and belonging are still needed.

A practical way to test choir’s incremental contribution is to use active comparators that match key ingredients one at a time. For instance, dance or synchronized exercise can match interpersonal entrainment; breathing/relaxation groups can match paced respiration; and group therapy can match narrative work via words. Choir differs by integrating all three in a single practice: participants regulate through controlled exhalation and phonation, express indirectly through pre-existing lyrical narratives, and experience “being-with” through voice blending rather than competition or turn-taking. If outcomes improve most when these elements co-occur, this would support a choir-specific, mechanism-based account rather than a generic “group activity” explanation.

Clinical practice also needs clearer guidance. Many therapists are open to using choir as an adjunct to treatment, but they do not yet have standard protocols. One practical option is to place choir sessions before or after talk therapy, so that singing can serve as an emotional warm-up or an emotional integration phase. Treatment teams can also work with music professionals to choose suitable repertoire and rehearsal formats for trauma survivors. In this way, choir may become a regular part of the treatment plan instead of a loosely attached extra activity.

These open questions point to a need for interdisciplinary collaboration. Researchers and practitioners from psychotherapy, music education, and the social sciences all need to take part in this work. Through this kind of cooperation, we can gain a clearer picture of the specific role of choir in trauma recovery and apply it more effectively in real-world settings.

## 6 Conclusion

Choral singing is not just a simple way to vent emotions. It allows people to express feelings, sort through their experience, and reconnect with themselves and others in a safe group setting. For many trauma survivors, the singing voice opens a new narrative path, so that experiences that cannot be spoken can still be heard in another form. Shared breathing, blended vocal lines, and common goals in the choir help people feel that they are not alone.

If we see trauma as something that breaks a person’s life story apart, choir becomes a tool that can help them put this story back together. It allows pain to be heard, and it lets loneliness receive an answer in the harmony. Through this shared practice of sound, survivors can find new strength in the group and take concrete, real steps toward posttraumatic growth.
